# Supplementary material for: Solutes unmask differences in clustering versus phase separation of FET proteins
Source: Nat Commun. 2024 May 23;15:4408. doi: 10.1038/s41467-024-48775-3 (PMC11116469; doi:10.1038/s41467-024-48775-3)
Supplement: Supplementary file 1 — Supplementary Information [file 41467_2024_48775_MOESM1_ESM.docx]

**Supplementary Information**

**For**

**Solutes unmask differences in clustering versus phase separation of FET proteins**

Mrityunjoy Kar ^1^, Laura T. Vogel ^2,§^, Gaurav Chauhan ^3,§^, Suren Felekyan ^2^, Hannes Ausserwöger ^4^, Timothy J. Welsh ^4^, Furqan Dar ^3^, Anjana R. Kamath ^1^, Tuomas P. J. Knowles ^4^, Anthony A. Hyman ^1, *^, Claus A. M. Seidel ^2, *^, and Rohit V. Pappu ^3, *^

^1^ Max Planck Institute of Cell Biology and Genetics, 01307, Dresden, Germany

^2^ Department of Molecular Physical Chemistry, Heinrich Heine University, 40225, Düsseldorf, Germany

^3^ Department of Biomedical Engineering and Center for Biomolecular Condensates, Washington University in St. Louis, St. Louis, MO 63130, USA

^4^ Centre for Misfolding Diseases, Yusuf Hamied Department of Chemistry, University of Cambridge, CB2 1EW, Cambridge, UK

^§^Equal contributions; *E-Mail: [hyman@mpi-cbg.de](mailto:hyman@mpi-cbg.de), [cseidel@hhu.de](mailto:cseidel@hhu.de), [pappu@wustl.edu](mailto:pappu@wustl.edu)

**Data for Supplementary Figures**

All data for supplementary figures are available as part of the same Source Data file that that provides data for the main text figures. These data are available via the GitHub repository of the Pappu lab ( <https://github.com/Pappulab/Glutamate_vs_Chloride_Clustering/> ). The MD simulations data generated in this study have been deposited in the Zenodo database under accession code <https://doi.org/10.5281/zenodo.10593297>.

**
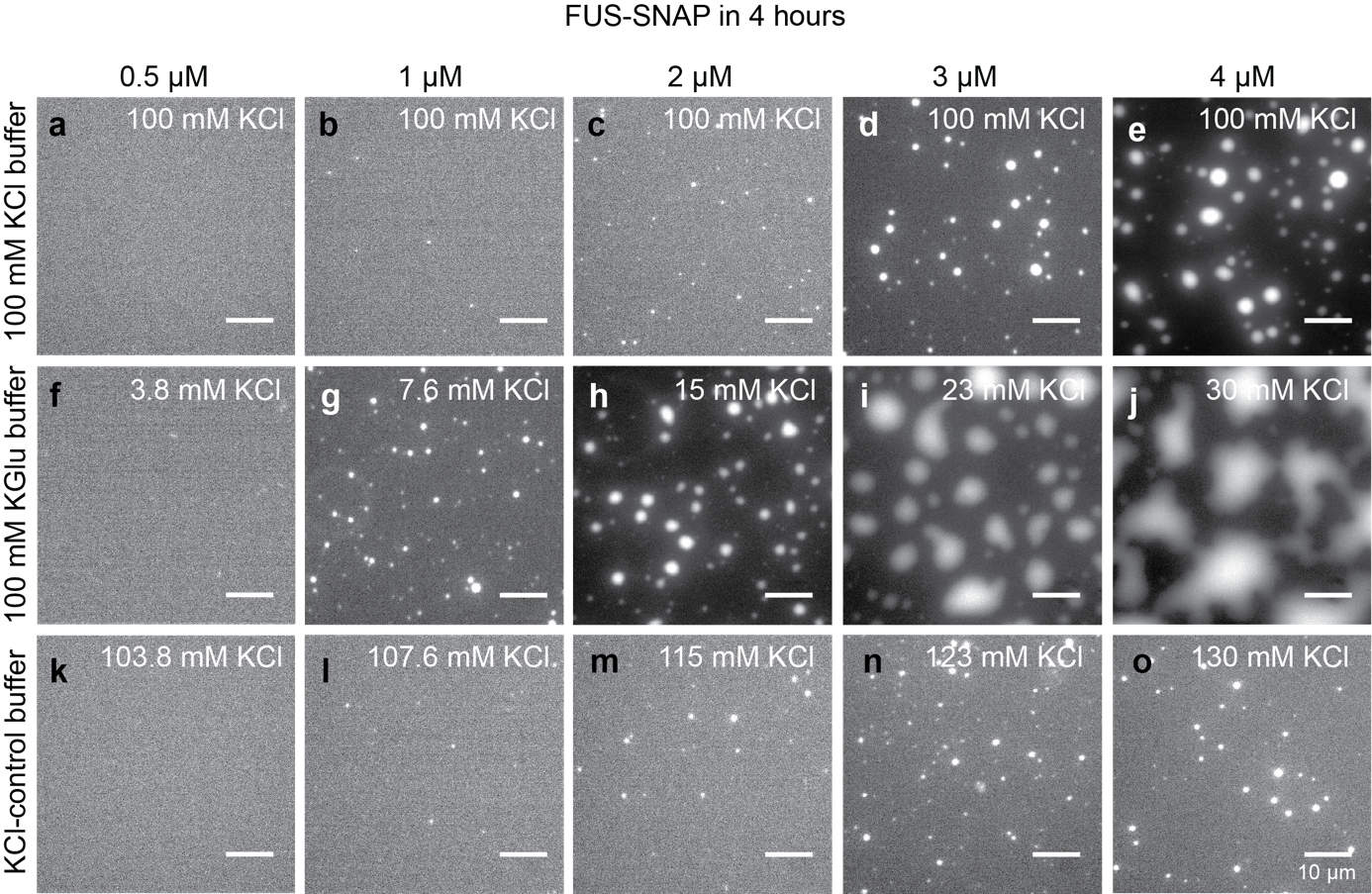
Supplementary Figure 1: Potassium Glutamate (KGlu) buffer has minimal influence on the driving forces for phase separation of FUS-SNAP, although the evolution of condensates is discernibly different**. (a)-(e) shows microscopy images collected at the 4-hour time point for solutions containing different concentrations of FUS-SNAP in 20 mM Tris.HCl, pH 7.4, with a final concentration of 100 mM KCl. Panels (f)-(j) show microscopy images collected at the 4-hour time point for solutions containing different concentrations of FUS-SNAP in 20 mM Tris.Glu, pH 7.4, with 100 mM KGlu, and panels (k)-(o) show microscopy images collected at the 4-hour time point for solutions containing different concentrations of FUS-SNAP in 20 mM Tris.HCl, pH 7.4, with 100 mM KCl. In both KGlu and KCl buffer, the residual KCl (< 30 mM) from FUS-SNAP stock was added. For imaging purposes, 5% of the total mixture in each sample is made up of AlexaFluor 488 labeled FUS-SNAP. The total KCl concentration in the solution is marked on the panels. The scale bar in all panels corresponds to 10 µm.


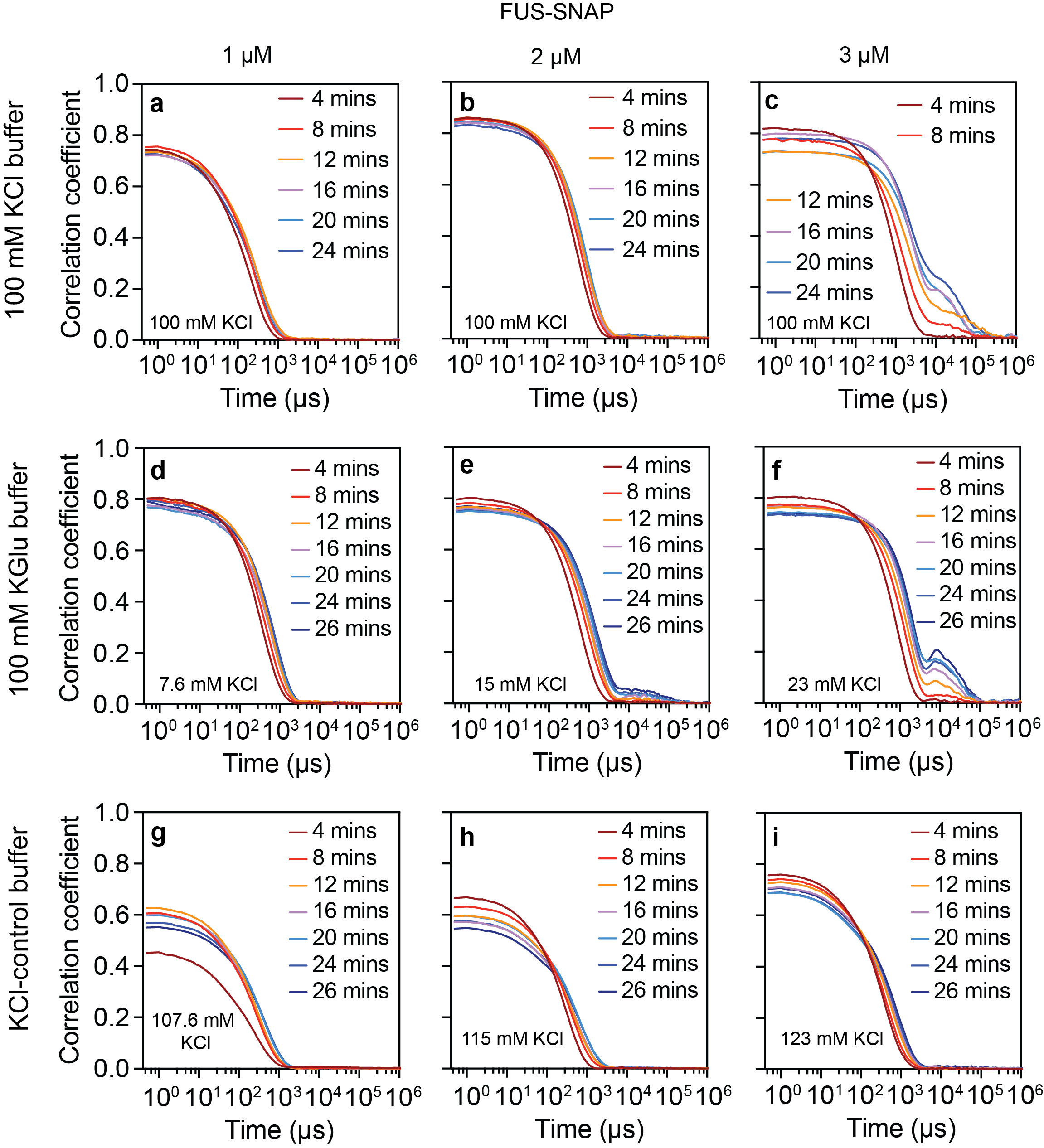


**Supplementary Figure 2:** **KGlu buffer minimally affects the driving forces for phase separation of FUS-SNAP, although the evolution of condensates is discernibly different**. (a)-(c) The correlation function from DLS of solutions containing different concentrations of FUS-SNAP, 1 μM (a), 2 μM (b), and 3 μM (c) in 20 mM Tris.HCl, pH 7.4, with a final concentration of 100 mM KCl. Panels (d), (e), and (f) show the correlation functions of solutions containing 1 μM, 2 μM, and 3 μM concentrations of FUS-SNAP, respectively, in 20 mM Tris.Glu, pH 7.4, with 100 mM KGlu. Panels (g), (h), and (i) show the correlation functions of solutions containing 1 μM, 2 μM, and 3 μM concentrations of FUS-SNAP, respectively, in 20 mM Tris.HCl, pH 7.4, with 100 mM KCl. The total concentration of KCl is marked on the panels. The correlation coefficient values indicate the abundance of clusters in the solutions. The time axis correlates with the size of the species; larger sizes require more time to decay, and vice versa.


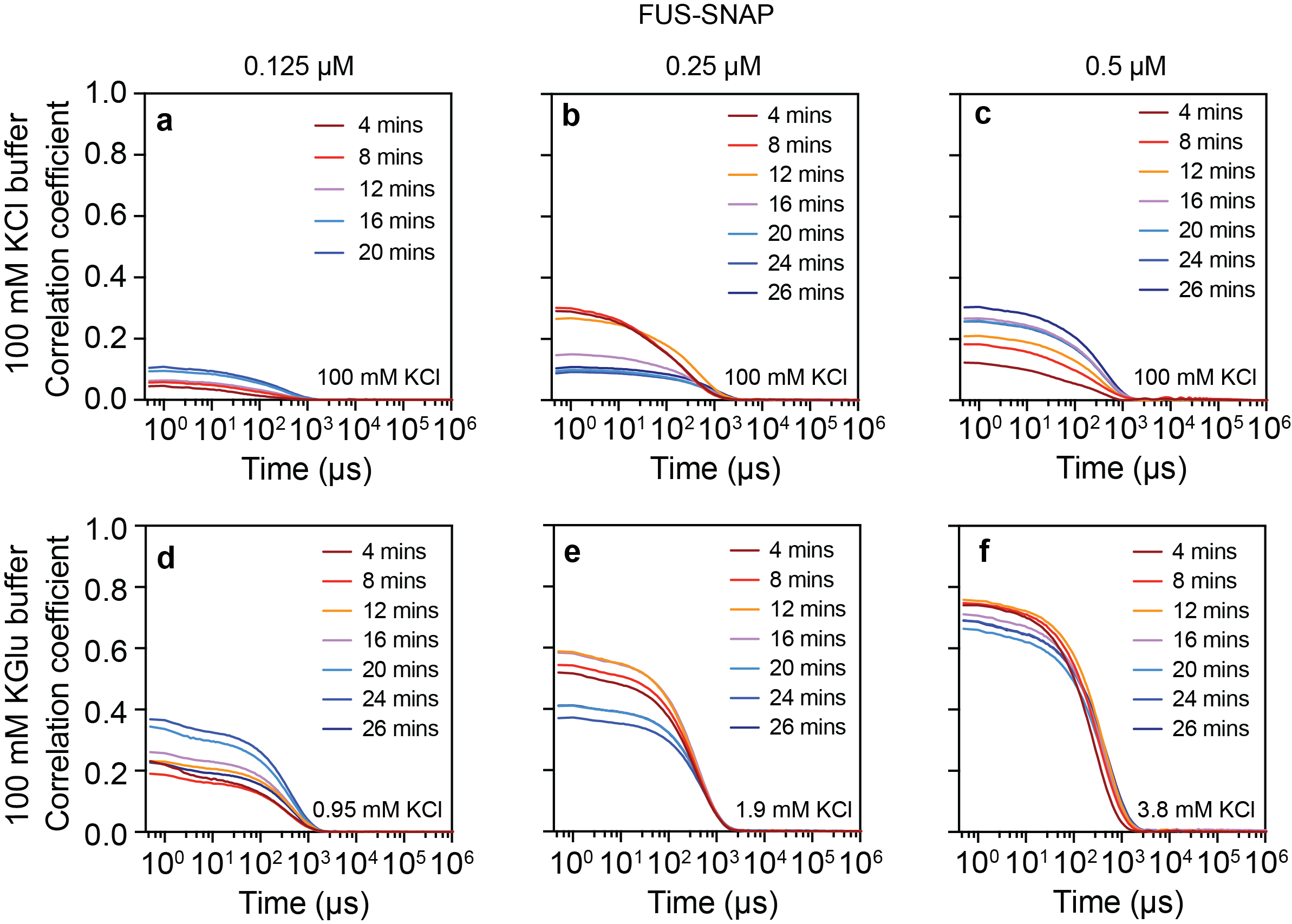


**Supplementary Figure 3:** **At lower sub-saturation concentrations, the cluster formation of FUS-SNAP in KGlu buffer is significantly higher compared to KCl buffer.** (a)-(c) The correlation function from the dynamic light scattering of solutions containing different concentrations of FUS-SNAP, 0.125 μM (a), 0.25 μM (b), and 0.5 μM (c) in 20 mM Tris.HCl, pH 7.4, with a final concentration of 100 mM KCl. Panels (d), (e), and (f) show the correlation functions of solutions containing 0.125 μM, 0.25 μM, and 0.5 μM concentrations of FUS-SNAP, respectively, in 20 mM Tris.Glu, pH 7.4, with 100 mM KGlu.


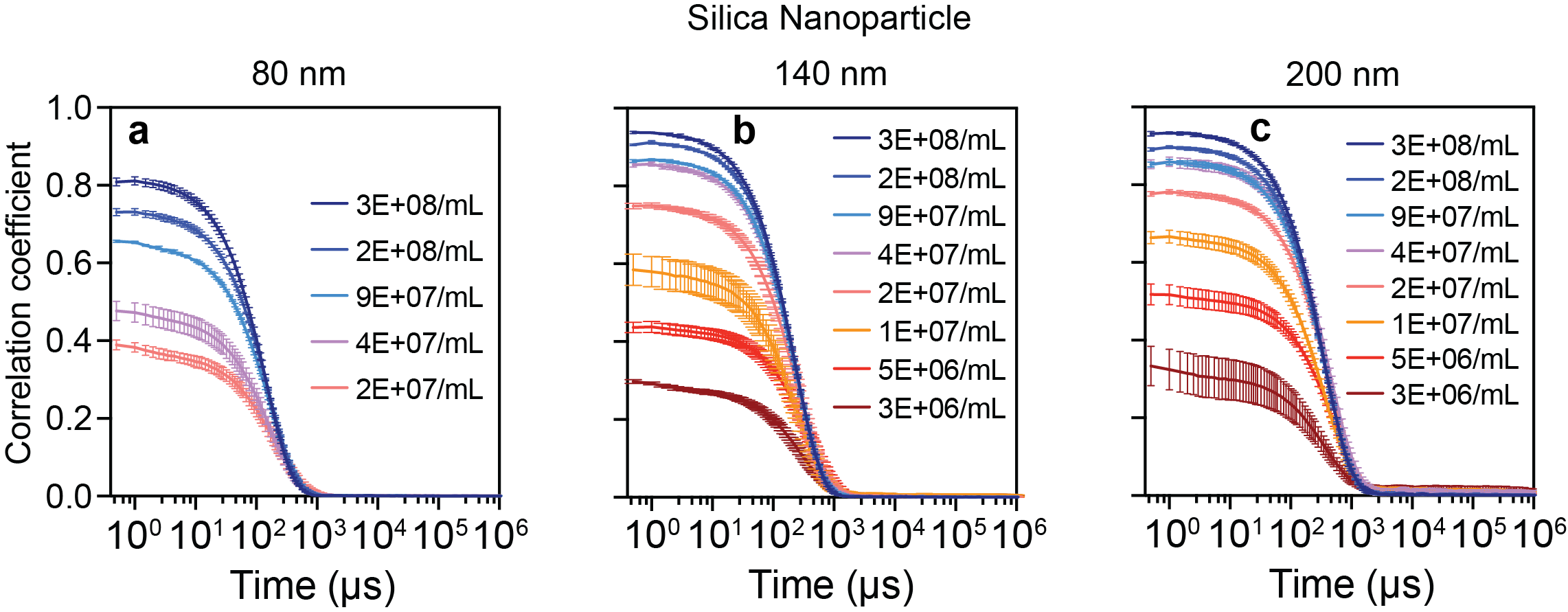


**Supplementary Figure 4:** **Calibration curves to interpret measured correlation coefficients from DLS.** Data were collected at different concentrations of monodisperse silica nanoparticles of various sizes. (a)-(c) The correlation function from the dynamic light scattering of solutions containing different concentrations of silica nanoparticles of the following sizes: 80 nm (a), 140 nm (b), and 200 nm (c) in 20 mM Tris.HCl, pH 7.4. The correlation coefficient value indicates the known concentration of silica nanoparticles in the solutions. The coefficient value increases with increasing concentration of silica nanoparticles. The time axis correlates with the size of the species; the larger size requires more time to decay, and vice versa. n=3 independent samples were used for all the measurements, and error bars are presented as mean values +/- SD.


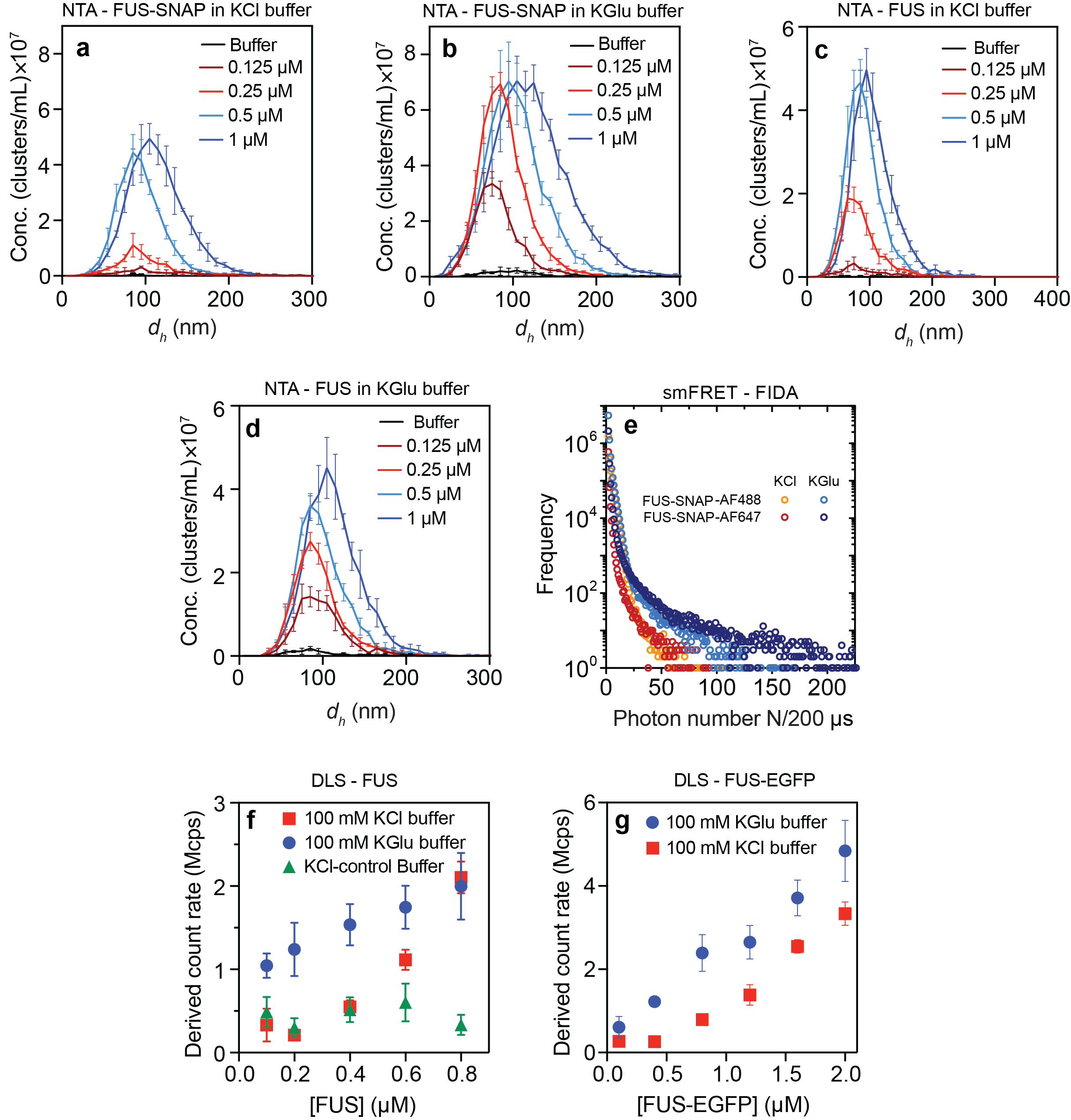


**Supplementary Figure 5:** **KGlu buffer enhances cluster formation of FUS-SNAP and FUS when compared to KCl-based buffer**. The distribution of cluster sizes measured using NTA of (a) FUS-SNAP in 100 mM KGlu buffer, (b) FUS-SNAP in 100 mM KCl buffer, (c) FUS in 100 mM KCl buffer, and (d) FUS in 100 mM KGlu buffer (d). n=3 independent samples were used for all the measurements. (e) Fluorescence intensity distribution analysis of single-molecule FRET (smFRET) measurements with 200 pM FUS-SNAP-AF488 as the donor and 200 pM FUS-SNAP-AF647 as acceptor shows more pronounced tailing towards higher photon numbers for donor/acceptor in KGlu (light blue/dark blue) than in KCl (orange/red). (f)-(g) DLS data show the derived count rate of FUS (f) and FUS-EGFP (g) in different buffers. n=3 independent samples were used for all the measurements, and error bars are presented as mean values ± SD.


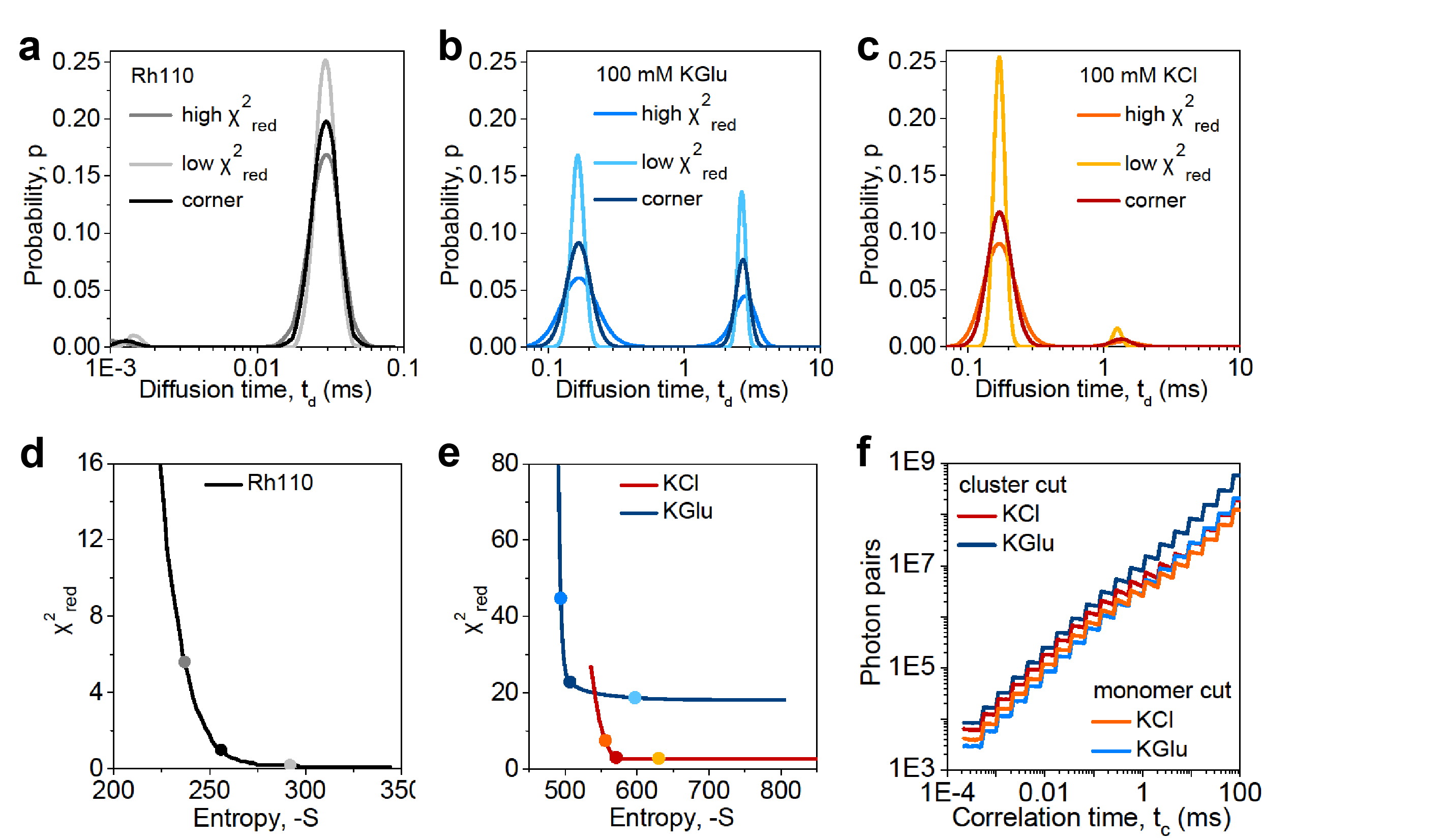


**Supplementary Figure 6: Results from analysis of cluster size distributions obtained using single-molecule data and the MEME.** (a-c) MEM translational diffusion time distributions for single molecule concentrations of (a) Rh110 and FUS-SNAP-AF488 in (b) glutamate and (c) chloride buffers showing the χ^2^-dependency of the distribution width. (d-e) L-curves are computed for each displayed distribution of the (d) dye reference and (e) FUS-SNAP-AF488 measurements in both buffer. (f) The photon pair histograms for the SMD of FUS-SNAP-AF488 accordingly to the applied cluster cut and monomer cut demonstrate similar statistics for all data sets thus ensuring the comparability of the fit quality.


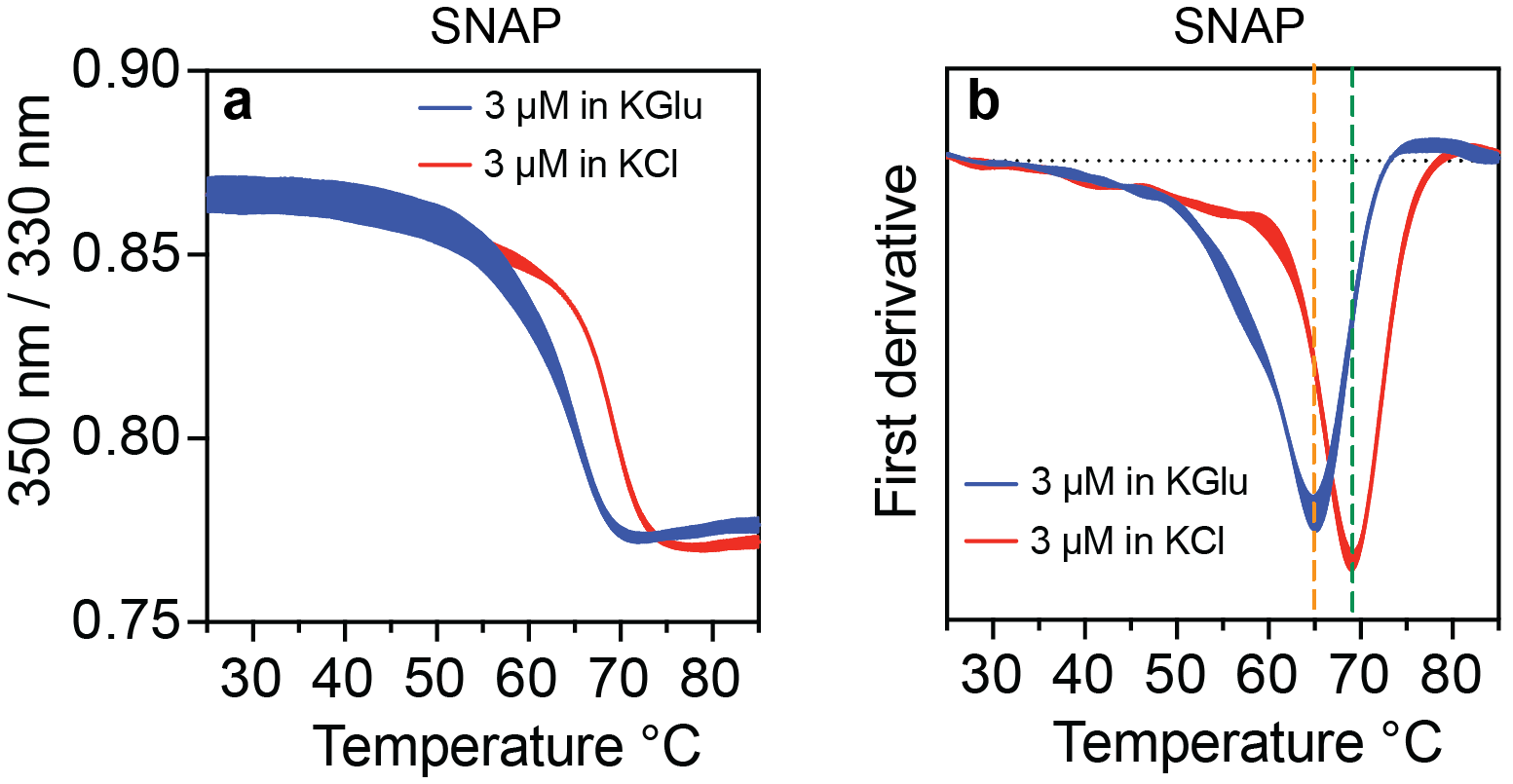


**Supplementary Figure 7: Analysis of data from nanoDSF measurements** (a) NanoDSF data show the 350 nm/330 nm ratio plotted against the temperature of SNAP in KCl and KGlu buffers. (b) The first derivative of data (a) plotted against temperature shows the apparent unfolding temperature of SNAP at 65°C and 69°C in KGlu and KCl buffers, respectively. n=3 independent samples were used for all the measurements, and error bars are presented as mean values ± SD.


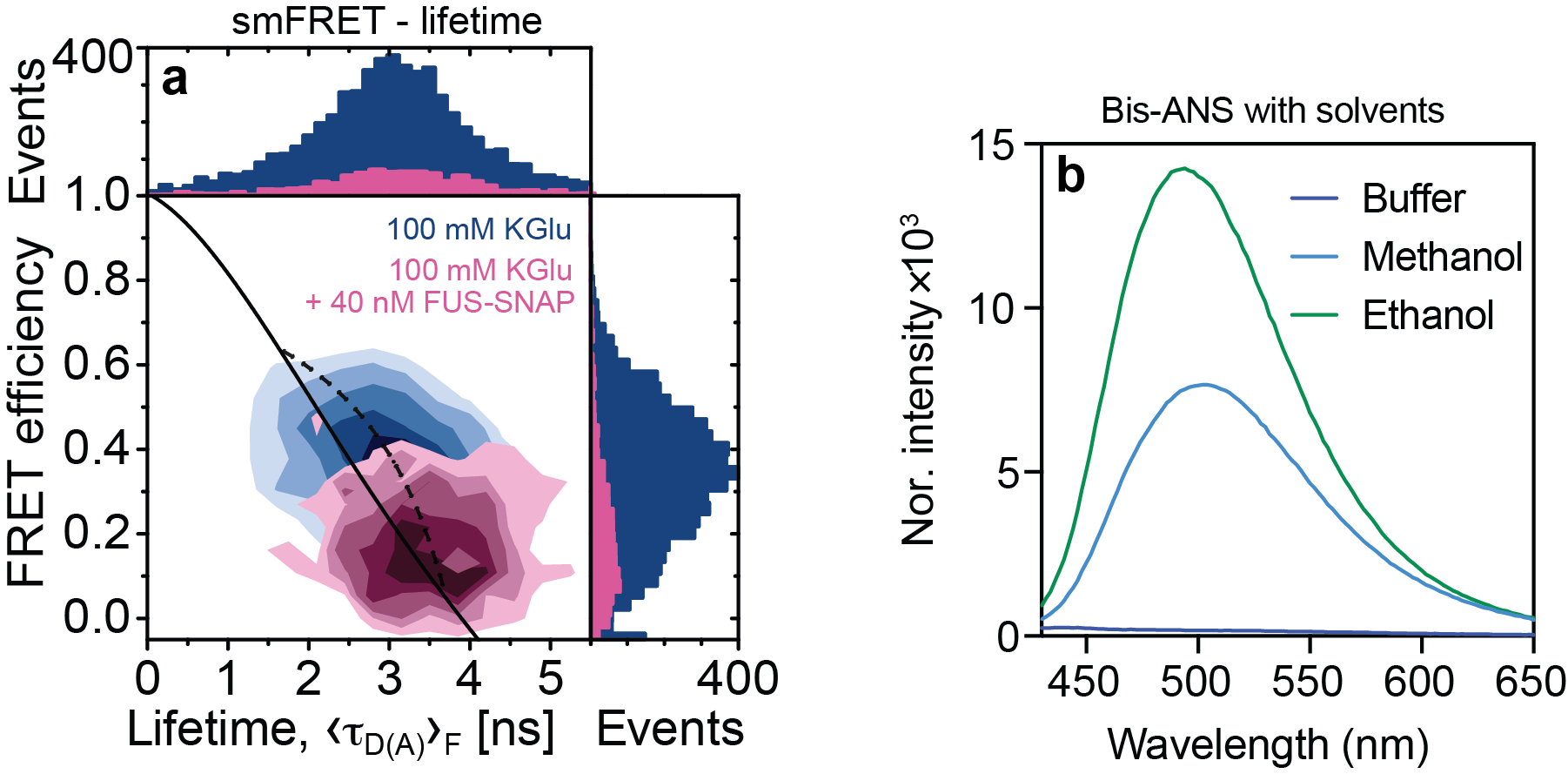


**Supplementary Figure 8: The FUS-SNAP clusters in KGlu buffer are reversible:** (a) Under single-molecule conditions, adding upon the 400 pM donor (AF488) and acceptor (AF647) labeled FUS-SNAP (measurement time 8 hours) 40 nM unlabeled FUS-SNAP, we observe a significant decrease in FRET efficiency, demonstrating the reversibility of FUS association on the millisecond time scale limited by translational diffusion. (b) The control experiments were where 2 µM Bis-ANS was mixed with buffer, methanol, and Ethanol.


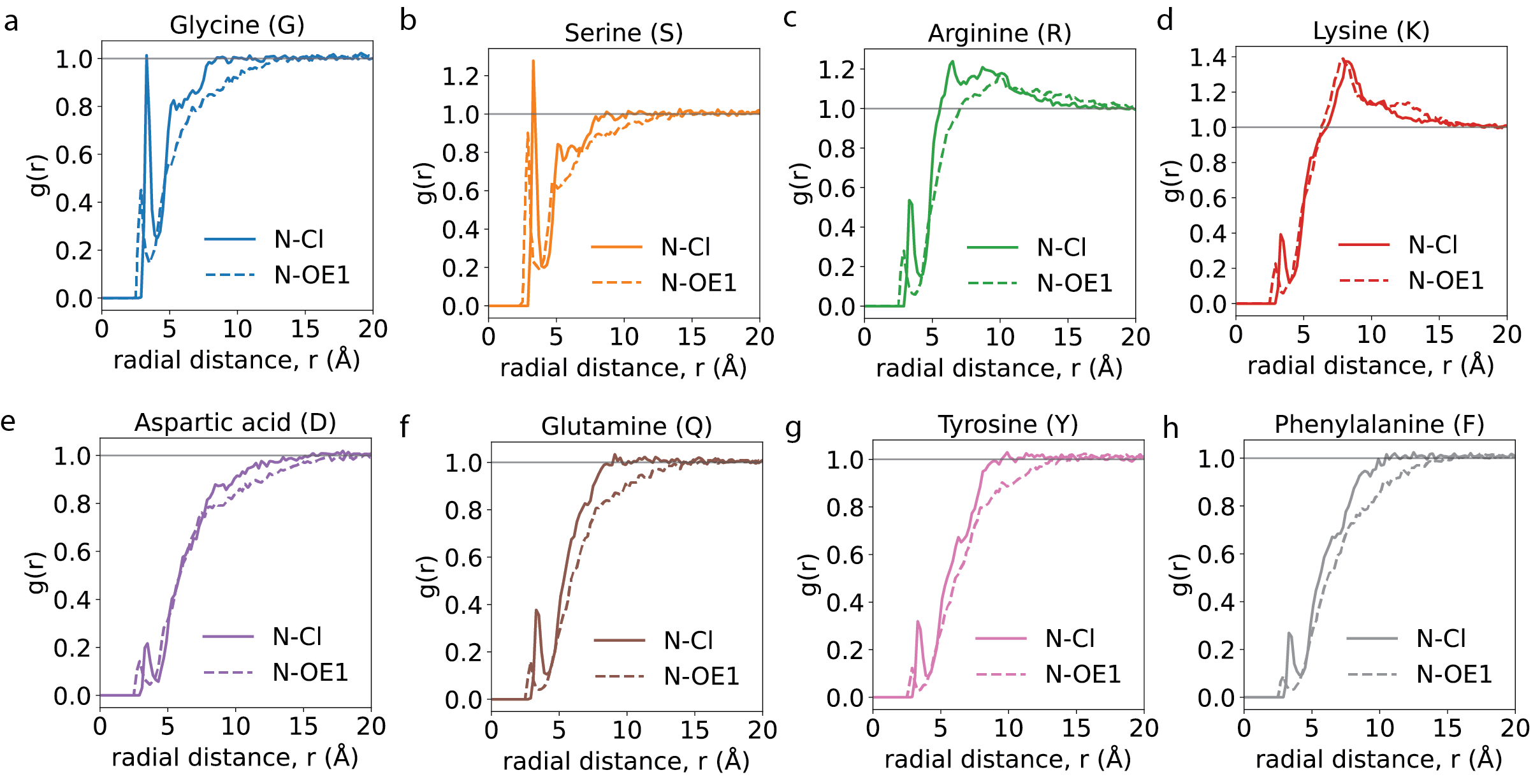


**Supplementary Figure 9:** **Site-site radial distribution functions, g(r) around backbone amide nitrogen atoms.** These quantify the relative probability, with respect to an ideal gas prior, of finding Cl^-^ atoms (solid curve) or the *sp*^2^ oxygen OE1 of glutamate around the amide nitrogen of the backbone for different capped amino acids. Data are shown for the g(r) of Cl- (solid curve) and OE1 atom of glutamate (dashed curve) around the backbone amide nitrogen of (a) Gly, (b) Ser, (c) Arg, (d) Lys, (e) Asp, (f) Gln, (g) Tyr, and (h) Phe. For Gly, Ser, and to a smaller extent for Gln, Tyr and Phe, we observed differences in the form of lower occupancies of OE1 when compared to Cl^-^, suggesting preferential exclusion. The differences in the occupancies around the backbone nitrogen originate, in part, from the differences in sidechain length and the type of functional group that makes up the sidechain. Note that the preferential exclusion extends to a spatial range of 10 – 12 Å. However, for Arg, Lys, and Asp, electrostatic considerations weaken the differences between the computed g(r) of the anions around the backbone amides. The electrostatic interactions refer to the attractions of the anions to sidechains of Arg and Lys, and repulsions from the sidechain of Asp. Due to covalent connectivity, these electrostatic effects influence the occupancies around the backbone nitrogen atoms, thereby weakening the differences between the solution anions.


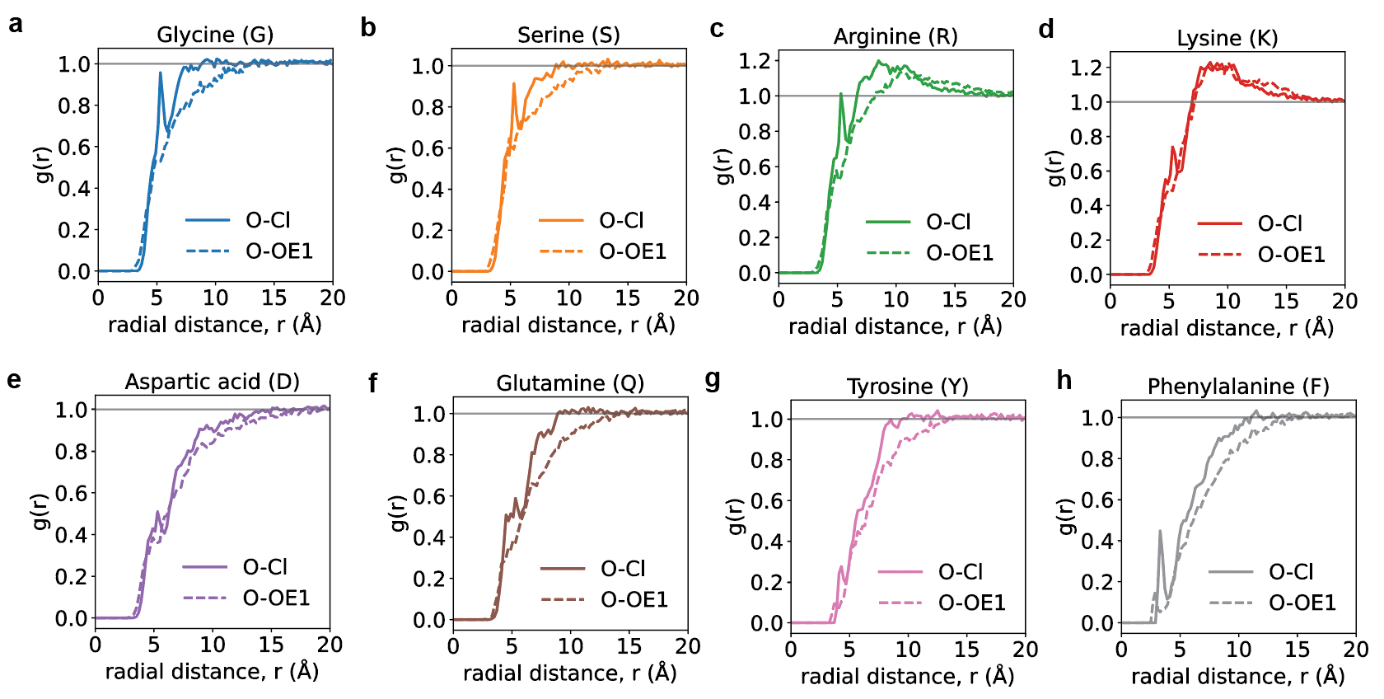


**Supplementary Figure 10:** **Site-site radial distribution functions, g(r) around backbone carbonyl oxygen atoms.** These quantify the relative probability, with respect to an ideal gas prior, of finding Cl^-^ atoms (solid curve) or the *sp*^2^ oxygen OE1 of glutamate around the carbonyl oxygen of the backbone for different capped amino acids. Data are shown for the g(r) of Cl- (solid curve) and OE1 atom of glutamate (dashed curve) around the backbone carbonyl oxygen of (a) Gly, (b) Ser, (c) Arg, (d) Lys, (e) Asp, (f) Gln, (g) Tyr, and (h) Phe.


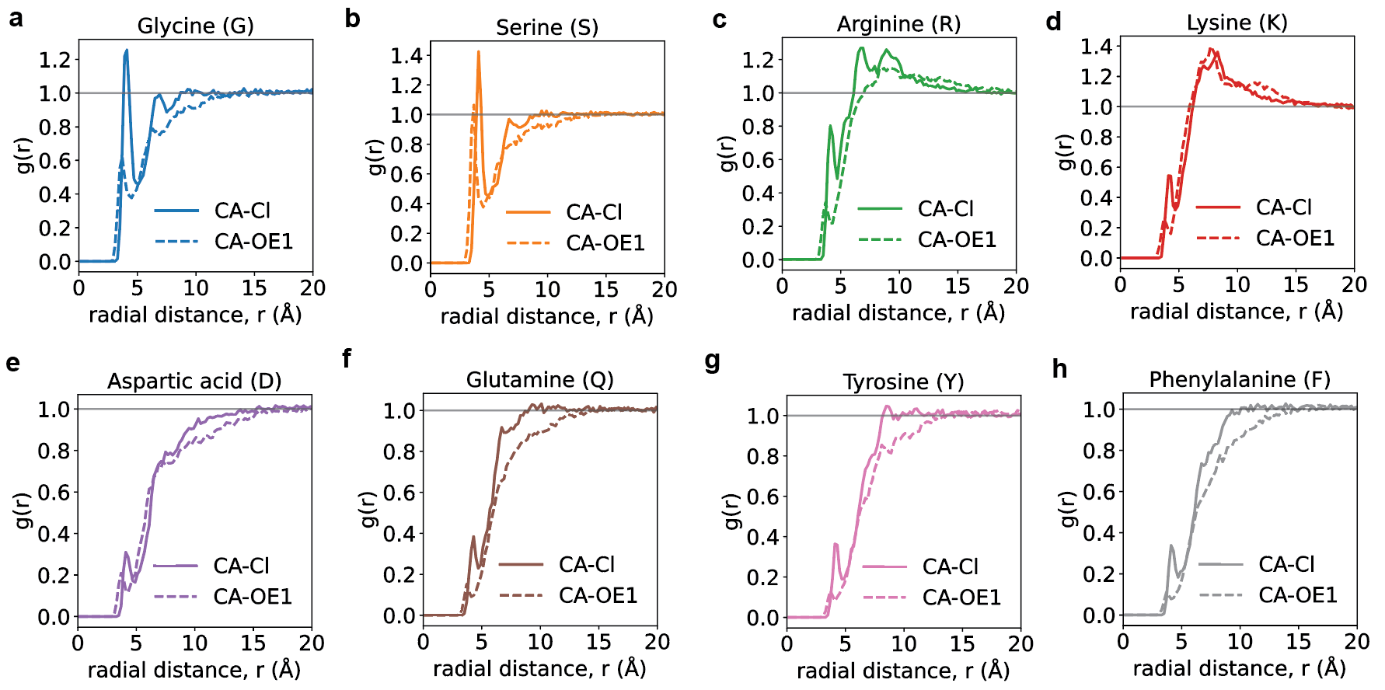


**Supplementary Figure 11:** **Site-site radial distribution functions, g(r) around the central (alpha) carbon atoms.** These quantify the relative probability, with respect to an ideal gas prior, of finding Cl^-^ atoms (solid curve) or the *sp*^2^ oxygen OE1 of glutamate around the alpha carbon of the backbone for different capped amino acids. Data are shown for the g(r) of Cl- (solid curve) and OE1 atom of glutamate (dashed curve) around the backbone central (alpha) carbon atom of (a) Gly, (b) Ser, (c) Arg, (d) Lys, (e) Asp, (f) Gln, (g) Tyr, and (h) Phe. While the preferential exclusion of the glutamate OE1 is pronounced around the central carbon of Gly and the alpha carbon of Ser, the differences are less pronounced around the alpha carbon of Gln, Tyr, and Phe. A combination of sidechain length and the site-specific strong versus weak interactions with the sidechain amides influence the occupancies around the alpha carbon atom. The influence of occupancies around the sidechain functional groups, summarized in **Supplementary Fig. S8**, influences the differences observed in the occupancies of Cl^-^ and OE1 of glutamate around the alpha carbon atoms of Lys versus Arg versus Asp.


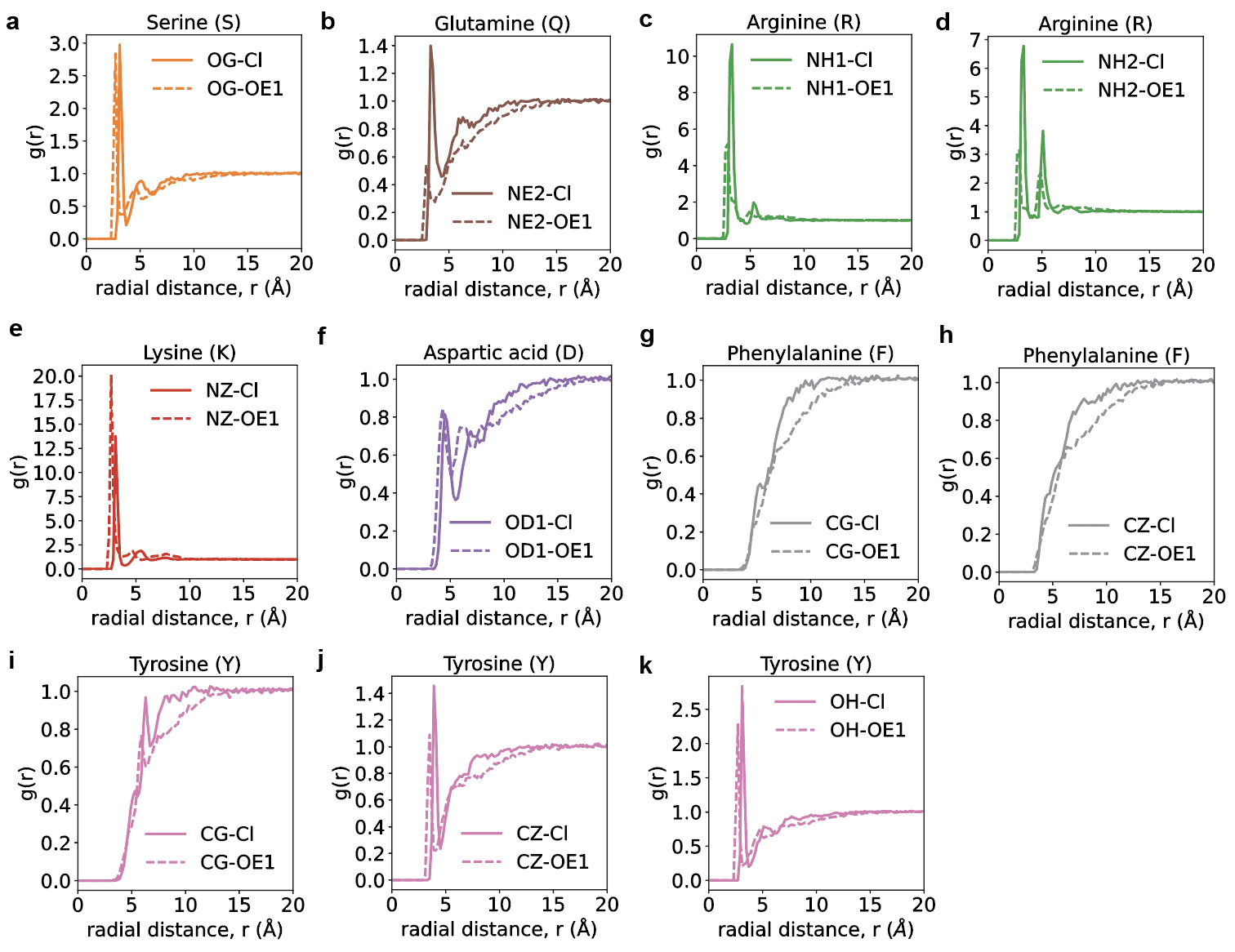


**Supplementary Figure 12:** **Site-site radial distribution functions, g(r) of the solution anions around specific sites of functional groups of sidechains.** These quantify the relative probability, with respect to an ideal gas prior, of finding Cl^-^ atoms (solid curves) or the *sp*^2^ oxygen OE1 of glutamate (dashed curves). (a) Comparative g(r) profiles of Cl^-^ versus the epsilon 1 (OE1) oxygen of glutamate around the gamma oxygen of the hydroxyl group of Ser. The OE1 is more proximal than the Cl^-^, and this has to do with the differences in the sizes of the atoms. However, the strengths of the interactions, quantified in terms of the heights of the g(r) profiles, are equivalent. Cl^-^ and the glutamate OE1 interact favorably and equivalently with the hydroxyl of Ser sidechains. (b) Comparative g(r) profiles of Cl^-^ versus the epsilon 1 (OE1) oxygen of glutamate around the epsilon 2 nitrogen, the hydrogen bond donor, of the primary amide of Gln. Again, the leftward shift of the OE1 profile has to do with the smaller size vis-à-vis Cl^-^. The preferential exclusion of glutamate from amides versus the preferential interaction of Cl^-^ with these sites is evident from the comparisons of the g(r) profiles. (c) and (d) In general, the interactions of Cl^-^ are stronger with the guanido group of Arg as evidenced by the clear differences in the site-site g(r) profiles of Cl^-^ and OE1 around the eta nitrogen atoms within the guanido moieties. (e) The interactions of OE1 are modestly stronger with the amine of lysine when compared to those of Cl^-^. (f) Electrostatic repulsions contribute to the roughly equivalent weakening of the interactions of both Cl^-^ and OE1 around the delta oxygen of Asp. (g) and (h) The interactions of OE1 are modestly weaker than the Cl^-^ around the gamma and zeta carbons of the aromatic ring in Phe. (i) and (j) Similar to Phe, the interactions of OE1 are modestly weaker than the Cl^-^ around the gamma and zeta carbons of the aromatic ring in Tyr. However, the presence of OH group on the zeta carbon leads to changes in g(r) when compared to Phe. (k) Comparative g(r) profiles of Cl^-^ versus the epsilon 1 (OE1) oxygen of glutamate around the OH group on the zeta carbon. Both Cl^-^ and glutamate ions interact favorably with the OH group with modestly weaker interactions for the glutamate.

**Supplementary Table 1: Glutamate concentrations in different cell types**

| *E. coli* ^3^ | ~96 mM ^3^ |
| --- | --- |
| Budding yeast *Saccharomyces cerevisiae* | ~ 75 mM ^4^ |
| Mouse kidney (iBMK) epithelial cells | ~64 mM ^5^ |
| Synaptic vesicles | ~100 mM ^6^ |
| Cytoplasm of neurons | ~5-10 mM ^7^ |
| Mammalian cancer cell line (glioblastoma) | ~20 mM ^8^ |

**Supplementary Table 2: FRET correction parameters**

| **Correction parameter ^9^** | | **FRET pair** |
| --- | --- | --- |
| **donor** | **D** | **FUS-SNAP-AF488** |
| **acceptor** | **A** | **FUS-SNAP-AF647** |
| *crosstalk* | *α* | 0.010 |
| *direct excitation* |  | 0.020 |
| *detection efficiency ratio* | $g_{G\left\vert D\right.}/g_{R\left\vert A\right.}$ | 0.800 |
| *donor fluorescence quantum yield* | $\Phi_{F,D(0)}$ | 0.800 |
| *acceptor fluorescence quantum yield* | *$\Phi_{F,A}$* | 0.330 |
| *acceptor-donor intensity ratio* | *δ* | 1.000 |
| *green background [kHz]* | *B_g_* | 1.650 |
| *red background [kHz]* | *B_r_* | 0.707 |
| *yellow background [kHz]* | *B_y_* | 0.672 |

**Supplementary Table 3: FCS fit parameters of FUS monomers using eq. 6a**

| **Fit parameter** | | | **Rh110** | **100 mM KCl** | **100 mM KGlu** |
| --- | --- | --- | --- | --- | --- |
| *χ^2^* | *chi-squared* | 1.732 | | 1.603 | 2.873 |
| G_0_ | *correlation offset* | 0.994 | | 0.993 | 0.994 |
| N | *number of molecules in focus* | 0.780 | | 0.822 | 0.816 |
| t_d,dye_ | *diffusion time of free dye impurities (global for three data sets) [ms]* | 0.042 | | 0.042 | 0.042 |
| z_0,1_/ω_0,1_ | *focus ratio for t_d1,global_* | 8.616 | | 9.729 | 6.211 |
| t_d,mo_ | *diffusion time of the monomer [ms]* | 0.187 | | 0.189 | 0.201 |
| z_0,2_/ω_0,2_ | *focus ratio for t_d2_* | 6.432 | | 11.33 | 16.09 |
| f | *amplitude for t_d1,global_* | 0.000 | | 0.186 | 0.209 |
| A | *amplitude for bunching term* | 0.046 | | 0.115 | 0.156 |
| t_A_ | *bunching time (global) [ms]* | 0.004 | | 0.418 | 0.367 |

**Supplementary Table 4: FCS fit parameters of FUS clusters using eq. 6b**

| **Fit parameter** | | | **100 mM KCl** | | **100 mM KGlu** | |
| --- | --- | --- | --- | --- | --- | --- |
| *χ^2^* | *chi-squared* | 1.365 | | 40.67 | |  |
| G_0_ | *correlation offset* | 1.000 | | 0.999 | |  |
| N | *number of molecules in focus* | 0.877 | | 0.872 | |  |
| t_d,mo_ | *diffusion time of the monomer (global for both data sets) [ms]* | 0.214 | | 0.214 | |  |
| z_0,1_/ω_0,1_ | *focus ratio for t_d1,global_* | 1.983 | | 2.090 | |  |
| t_d,cl_ | *diffusion time of FUS clusters [ms]* | 1.055 | | 2.260 | |  |
| z_0,2_/ω_0,2_ | *focus ratio for t_d2_* | 6.125 | | 598.4 | |  |
| f | *amplitude for t_d,mo_* | 0.707 | | 0.718 | |  |
| A | *amplitude for bunching term* | 0.076 | | 0.089 | |  |
| t_A_ | *bunching time (global) [ms]* | 0.003 | | 0.003 | |  |

**Supplementary Note 1**

**Materials for**

**Solutes unmask differences in clustering versus phase separation of FET proteins**

Mrityunjoy Kar ^1^, Laura T. Vogel ^2,§^, Gaurav Chauhan ^3,§^, Suren Felekyan ^2^, Hannes Ausserwöger ^4^, Timothy J. Welsh ^4^, Furqan Dar ^3^, Anjana R. Kamath ^1^, Tuomas P. J. Knowles ^4^, Anthony A. Hyman ^1, *^, Claus A. M. Seidel ^2, *^, and Rohit V. Pappu ^3, *^

^1^ Max Planck Institute of Cell Biology and Genetics, 01307, Dresden, Germany

^2^ Department of Molecular Physical Chemistry, Heinrich Heine University, 40225, Düsseldorf, Germany

^3^ Department of Biomedical Engineering and Center for Biomolecular Condensates, Washington University in St. Louis, St. Louis, MO 63130, USA

^4^ Centre for Misfolding Diseases, Yusuf Hamied Department of Chemistry, University of Cambridge, CB2 1EW, Cambridge, UK

^§^Equal contributions; *E-Mail: [hyman@mpi-cbg.de](mailto:hyman@mpi-cbg.de), [cseidel@hhu.de](mailto:cseidel@hhu.de), [pappu@wustl.edu](mailto:pappu@wustl.edu)

**Materials**

**List of reagents, sources, and vendor identifiers if any**

| **REAGENTS** | **SOURCE** | **IDENTIFIER** |
| --- | --- | --- |
| **CHEMICALS** | | |
| TRIS | Carl Roth Germany | 77-86-1 |
| Potassium Chloride (KCl) | Merck Germany | 7447-40-7 |
| Potassium Glutamate (KGlu) | Merck Germany | 6382-01-0 |
| Glutamic Acid | Merck Germany | 56-86-0 |
| Glycerol | VWR chemicals | 56-81-5 |
| cOmplete^TM^ | Roche Germany | 11697498001 |
| Imidazole | Merck Germany | 288-32-4 |
| DTT | Alfa Aesar Germany | 578-51-7 |
| Maltose | Merck Germany | 6363-53-7 |
| Hydrochloric Acid | Merck Germany | 7647-01-0 |
| Bis-ANS | Merck Germany | 65664-81-5 |
| Nile Red | Merck Germany | 7385-67-3 |
| **Bacterial and Virus Strains** |  |  |
| Sf9 cells | Expression Systems | Cat#94-001F |
| **Recombinant proteins** |  |  |
| FUS-EGFP | Wang et. al. 2018 ^1^ | TH1204 |
| FUS-SNAP | Wang et. al. 2018 ^1^ | TH0901 |
| FUS | Wang et. al. 2018 ^1^ | TH0901 |
| FUS (RBD, 24R-K) | Wang et. al. 2018 ^1^ | TH1149 |
| FUS (RBD, 24R-G) | Wang et. al. 2018 ^1^ | TH1006 |
| FUS (RBD, 10D/4E -G) | Kar et. al. 2022 ^2^ | TH1740 |
| FUS (PLD,10Y-S) | Kar et. al. 2022 ^2^ | TH1427 |
| FUS (PLD, 18Y-S) | Kar et. al. 2022 ^2^ | TH1815 |
| FUS (PLD, 27Y-S) | Wang et. al. 2018 ^1^ | TH0992 |
| FUS (RBD, 6F-G) | Kar et. al. 2022 ^2^ | TH1917 |
| FUS (RBD, 6Y-S) | Kar et. al. 2022 ^2^ | TH1918 |
| TAF15-SNAP | Wang et. al. 2018 ^1^ | TH1203 |
| EWSR1-SNAP | Wang et. al. 2018 ^1^ | TH1276 |

**Supplementary References**

1. Wang J, Choi J-M, Holehouse AS, Lee HO, Zhang X, Jahnel M*, et al.* A Molecular Grammar Governing the Driving Forces for Phase Separation of Prion-like RNA Binding Proteins. *Cell* 2018, **174**(3)**:** 688-699. e616.

2. Kar M, Dar F, Welsh TJ, Vogel LT, Kühnemuth R, Majumdar A*, et al.* Phase-separating RNA-binding proteins form heterogeneous distributions of clusters in subsaturated solutions. *Proceedings of the National Academy of Sciences* 2022, **119**(28)**:** e2202222119.

3. Bennett BD, Kimball EH, Gao M, Osterhout R, Van Dien SJ, Rabinowitz JD. Absolute metabolite concentrations and implied enzyme active site occupancy in Escherichia coli. *Nature Chemical Biology* 2009, **5**(8)**:** 593-599.

4. van Eunen K, Bouwman J, Daran-Lapujade P, Postmus J, Canelas AB, Mensonides FIC*, et al.* Measuring enzyme activities under standardized in vivo-like conditions for systems biology. *The FEBS Journal* 2010, **277**(3)**:** 749-760.

5. Park JO, Rubin SA, Xu Y-F, Amador-Noguez D, Fan J, Shlomi T*, et al.* Metabolite concentrations, fluxes and free energies imply efficient enzyme usage. *Nature Chemical Biology* 2016, **12**(7)**:** 482-489.

6. Burger PM, Mehl E, Cameron PL, Maycox PR, Baumert M, Lottspeich F*, et al.* Synaptic vesicles immunoisolated from rat cerebral cortex contain high levels of glutamate. *Neuron* 1989, **3**(6)**:** 715-720.

7. Featherstone DE. Intercellular Glutamate Signaling in the Nervous System and Beyond. *ACS Chemical Neuroscience* 2010, **1**(1)**:** 4-12.

8. DeBerardinis RJ, Mancuso A, Daikhin E, Nissim I, Yudkoff M, Wehrli S*, et al.* Beyond aerobic glycolysis: Transformed cells can engage in glutamine metabolism that exceeds the requirement for protein and nucleotide synthesis. *Proceedings of the National Academy of Sciences* 2007, **104**(49)**:** 19345-19350.

9. Kudryavtsev V, Sikor M, Kalinin S, Mokranjac D, Seidel CAM, Lamb DC. Combining MFD and PIE for Accurate Single-Pair Förster Resonance Energy Transfer Measurements. *ChemPhysChem* 2012, **13**(4)**:** 1060-1078.
