# Supplementary material for: Solutes unmask differences in clustering versus phase separation of FET proteins
Source: Nat Commun. 2024 May 23;15:4408. doi: 10.1038/s41467-024-48775-3 (PMC11116469; doi:10.1038/s41467-024-48775-3)
Supplement: Supplementary file 2 — Description of Additional Supplementary Files [file 41467_2024_48775_MOESM2_ESM.docx]

**Description of Additional Supplementary Files**

**For**

**Solutes unmask differences in clustering versus phase separation of FET proteins**

Mrityunjoy Kar ^1^, Laura T. Vogel ^2,§^, Gaurav Chauhan ^3,§^, Suren Felekyan ^2^, Hannes Ausserwöger ^4^, Timothy J. Welsh ^4^, Furqan Dar ^3^, Anjana R. Kamath ^1^, Tuomas P. J. Knowles ^4^, Anthony A. Hyman ^1, *^, Claus A. M. Seidel ^2, *^, and Rohit V. Pappu ^3, *^

^1^ Max Planck Institute of Cell Biology and Genetics, 01307, Dresden, Germany

^2^ Department of Molecular Physical Chemistry, Heinrich Heine University, 40225, Düsseldorf, Germany

^3^ Department of Biomedical Engineering and Center for Biomolecular Condensates, Washington University in St. Louis, St. Louis, MO 63130, USA

^4^ Centre for Misfolding Diseases, Yusuf Hamied Department of Chemistry, University of Cambridge, CB2 1EW, Cambridge, UK

^§^Equal contributions; *E-Mail: [hyman@mpi-cbg.de](mailto:hyman@mpi-cbg.de), [cseidel@hhu.de](mailto:cseidel@hhu.de), [pappu@wustl.edu](mailto:pappu@wustl.edu)

**Captions for Supplementary Movies**

**Supplementary Movie 1:** Video from NTA measurements showing mesoscale clusters formed in 0.25 µM solutions of FUS-SNAP in the presence of glutamate.

**Supplementary Movie 2:** Video from NTA measurements showing mesoscale clusters formed in 0.5 µM solutions of FUS-SNAP in the presence of glutamate.

**Information contained in the Supplementary Data File:** This file provides details of the amino acid sequences of each of the FET constructs used in the study.
