# Supplementary material for: Solutes unmask differences in clustering versus phase separation of FET proteins
Source: Nat Commun. 2024 May 23;15:4408. doi: 10.1038/s41467-024-48775-3 (PMC11116469; doi:10.1038/s41467-024-48775-3)
Supplement: Supplementary file 3 — Supplementary Data [file 41467_2024_48775_MOESM3_ESM.docx]

**Supplementary Data**

**For**

**Solutes unmask differences in clustering versus phase separation of FET proteins**

Mrityunjoy Kar ^1^, Laura T. Vogel ^2,§^, Gaurav Chauhan ^3,§^, Suren Felekyan ^2^, Hannes Ausserwöger ^4^, Timothy J. Welsh ^4^, Furqan Dar ^3^, Anjana R. Kamath ^1^, Tuomas P. J. Knowles ^4^, Anthony A. Hyman ^1, *^, Claus A. M. Seidel ^2, *^, and Rohit V. Pappu ^3, *^

^1^ Max Planck Institute of Cell Biology and Genetics, 01307, Dresden, Germany

^2^ Department of Molecular Physical Chemistry, Heinrich Heine University, 40225, Düsseldorf, Germany

^3^ Department of Biomedical Engineering and Center for Biomolecular Condensates, Washington University in St. Louis, St. Louis, MO 63130, USA

^4^ Centre for Misfolding Diseases, Yusuf Hamied Department of Chemistry, University of Cambridge, CB2 1EW, Cambridge, UK

^§^Equal contributions; *E-Mail: [hyman@mpi-cbg.de](mailto:hyman@mpi-cbg.de), [cseidel@hhu.de](mailto:cseidel@hhu.de), [pappu@wustl.edu](mailto:pappu@wustl.edu)

**1. FUS-EGFP:** **This sequence includes full-length FUS (unshaded), a linker that is cleavable by a TEV protease (shaded in yellow), and the EGFP (shaded in green).**

MASNDYTQQATQSYGAYPTQPGQGYSQQSSQPYGQQSYSGYSQSTDTSGYGQSSYSSYGQSQNTGYGTQSTPQGYGSTGGYGSSQSSQSSYGQQSSYPGYGQQPAPSSTSGSYGSSSQSSSYGQPQSGSYSQQPSYGGQQQSYGQQQSYNPPQGYGQQNQYNSSSGGGGGGGGGGNYGQDQSSMSSGGGSGGGYGNQDQSGGGGSGGYGQQASDRGGRGRGGSGGGGGGGGGGYNRSSGGYEPRGRGGGRGGRGGMGGSDRGGFNKFGGPRDQGSRHDSEQDNSDNNTIFVQGLGENVTIESVADYFKQIGIIKTNKKTGQPMINLYTDRETGKKGEATVSFDDPPSAKAAIDWFDGKEFSGNPIKVSFATRRADFNRGGGNGRGGRGRGMGRGGYGGGGSGGGGRGGFPSGGGGGGGQQRAGDWKCPNPTCENMNFSWRNECNQCKAPKPDGPGGGPGGSHMGGNYGDDRRGGRGGYDRGGYRGRGGDRGGFRGGRGGGDRGGFGPGKMDSRGEHRQDRRERPYGAPGSSSGRENLYFQGMVSKGEELFTGVVPILVELDGDVNGHKFSVSGEGEGDATYGKLTLKFICTTGKLPVPWPTLVTTLTYGVQCFSRYPDHMKQHDFFKSAMPEGYVQERTIFFKDDGNYKTRAEVKFEGDTLVNRIELKGIDFKEDGNILGHKLEYNYNSHNVYIMADKQKNGIKVNFKIRHNIEDGSVQLADHYQQNTPIGDGPVLLPDNHYLSTQSALSKDPNEKRDHMVLLEFVTAAGITLGMDELYK

**2. FUS-SNAP: This sequence includes full-length FUS (unshaded), a linker that is cleavable by a TEV protease (shaded in yellow), and the SNAP (shaded in gray).**

MASNDYTQQATQSYGAYPTQPGQGYSQQSSQPYGQQSYSGYSQSTDTSGYGQSSYSSYGQSQNTGYGTQSTPQGYGSTGGYGSSQSSQSSYGQQSSYPGYGQQPAPSSTSGSYGSSSQSSSYGQPQSGSYSQQPSGGQQQSYGQQQSYNPPQGYGQQNQYNSSSGGGGGGGGGGNYGQDQSSMSSGGGSGGGYGNQDQSGGGGSGGYGQQASDRGGRGRGGSGGGGGGGGGGYNRSSGGYEPRGRGGGRGGRGGMGGSDRGGFNKFGGPRDQGSRHDSEQDNSDNNTIFVQGLGENVTIESVADYFKQIGIIKTNKKTGQPMINLYTDRETGKLKGEATVSFDDPPSAKAAIDWFDGKEFSGNPIKVSFATRRADFNRGGGNGRGGRGRGGPMGRGGYGGGGSGGGGRGGFPSGGGGGGGQQRAGDWKCPNPTCENMNFSWRNECNQCKAPKPDGPGGGPGGSHMGGNYGDDRRGGRGGYDRGGYRGRGGDRGGFRGGRGGGDRGGFGPGKMDSRGEHRQDRRERPYGAPGSSSGRENLYFQGMDKDCEMKRTTLDSPLGKLELSGCEQGLHRIIFLGKGTSAADAVEVPAPAAVLGGPEPLMQATAWLNAYFHQPEAIEEFPVPALHHPVFQQESFTRQVLWKLLKVVKFGEVISYSHLAALAGNPAATAAVKTALSGNPVPILIPCHRVVQGDLDVGGYEGGLAVKEWLLAHEGHRLGKPGLG

**3. FUS**

MASNDYTQQATQSYGAYPTQPGQGYSQQSSQPYGQQSYSGYSQSTDTSGYGQSSYSSYGQSQNTGYGTQSTPQGYGSTGGYGSSQSSQSSYGQQSSYPGYGQQPAPSSTSGSYGSSSQSSSYGQPQSGSYSQQPSYGGQQQSYGQQQSYNPPQGYGQQNQYNSSSGGGGGGGGGGNYGQDQSSMSSGGGSGGGYGNQDQSGGGGSGGYGQQASDRGGRGRGGSGGGGGGGGGGYNRSSGGYEPRGRGGGRGGRGGMGGSDRGGFNKFGGPRDQGSRHDSEQDNSDNNTIFVQGLGENVTIESVADYFKQIGIIKTNKKTGQPMINLYTDRETGKLKGEATVSFDDPPSAKAAIDWFDGKEFSGNPIKVSFATRRADFNRGGGNGRGGRGRGGPMGRGGYGGGGSGGGGRGGFPSGGGGGGGQQRAGDWKCPNPTCENMNFSWRNECNQCKAPKPDGPGGGPGGSHMGGNYGDDRRGGRGGYDRGGYRGRGGDRGGFRGGRGGGDRGGFGPGKMDSRGEHRQDRRERPY

**4. FUS(R-K): Full-length FUS with 24 Arg substituted to Lys in the RBD**

MASNDYTQQATQSYGAYPTQPGQGYSQQSSQPYGQQSYSGYSQSTDTSGYGQSSYSSYGQSQNTGYGTQSTPQGYGSTGGYGSSQSSQSSYGQQSSYPGYGQQPAPSSTSGSYGSSSQSSSYGQPQSGSYSQQPSYGGQQQSYGQQQSYNPPQGYGQQNQYNSSSGGGGGGGGGGNYGQDQSSMSSGGGSGGGYGNQDQSGGGGSGGYGQQASDKGGKGKGGSGGGGGGGGGGYNRSSGGYEPKGKGGGKGGKGGMGGSDKGGFNKFGGPRDQGSRHDSEQDNSDNNTIFVQGLGENVTIESVADYFKQIGIIKTNKKTGQPMINLYTDRETGKLKGEATVSFDDPPSAKAAIDWFDGKEFSGNPIKVSFATRRADFNKGGGNGKGGKGKGGPMGKGGYGGGGSGGGGKGGFPSGGGGGGGQQRAGDWKCPNPTCENMNFSWRNECNQCKAPKPDGPGGGPGGSHMGGNYGDDRKGGKGGYDKGGYKGKGGDKGGFRGGRGGGDRGGFGPGKMDSRGEHRQDRRERPY

**5. FUS(R-G): Full-length FUS with 24 Arg substituted to Gly in the RBD**

MASNDYTQQATQSYGAYPTQPGQGYSQQSSQPYGQQSYSGYSQSTDTSGYGQSSYSSYGQSQNTGYGTQSTPQGYGSTGGYGSSQSSQSSYGQQSSYPGYGQQPAPSSTSGSYGSSSQSSSYGQPQSGSYSQQPSYGGQQQSYGQQQSYNPPQGYGQQNQYNSSSGGGGGGGGGGNYGQDQSSMSSGGGSGGGYGNQDQSGGGGSGGYGQQASDGGGGGGGGSGGGGGGGGGGYNRSSGGYEPGGGGGGGGGGGGMGGSDGGGFNKFGGPRDQGSRHDSEQDNSDNNTIFVQGLGENVTIESVADYFKQIGIIKTNKKTGQPMINLYTDRETGKLKGEATVSFDDPPSAKAAIDWFDGKEFSGNPIKVSFATRRADFNGGGGNGGGGGGGGGPMGGGGYGGGGSGGGGGGGFPSGGGGGGGQQRAGDWKCPNPTCENMNFSWRNECNQCKAPKPDGPGGGPGGSHMGGNYGDDRGGGGGGYDGGGYGGGGGDGGGFGGGGGGGDGGGFGPGKMDSGGEHRQDRRERPY

**6. FUS-(10D/4E-G): Full-length FUS with 10 Asp and 4 Glu residues substituted to Gly in the RBD**

MASNDYTQQATQSYGAYPTQPGQGYSQQSSQPYGQQSYSGYSQSTDTSGYGQSSYSSYGQSQNTGYGTQSTPQGYGSTGGYGSSQSSQSSYGQQSSYPGYGQQPAPSSTSGSYGSSSQSSSYGQPQSGSYSQQPSYGGQQQSYGQQQSYNPPQGYGQQNQYNSSSGGGGGGGGGGNYGQDQSSMSSGGGSGGGYGNQDQSGGGGSGGYGQQASDRGGRGRGGSGGGGGGGGGGYNRSSGGYEPRGRGGGRGGRGGMGGSDRGGFNKFGGPRDQGSRHDSEQDNSDNNTIFVQGLGENVTIESVADYFKQIGIIKTNKKTGQPMINLYTDRETGKLKGEATVSFDDPPSAKAAIDWFDGKEFSGNPIKVSFATRRAGFNRGGGNGRGGRGRGGPMGRGGYGGGGSGGGGRGGFPSGGGGGGGQQRAGGWKCPNPTCGNMNFSWRNGCNQCKAPKPGGPGGGPGGSHMGGNYGGGRRGGRGGYGRGGYRGRGGGRGGFRGGRGGGGRGGFGPGKMGSRGGHRQGRRGRPY

**7. FUS(10Y-S): Full-length FUS with 10 Tyr residues substituted to Ser in the PLD**

MASNDYTQQATQSYGASPTQPGQGYSQQSSQPSGQQSYSGSSQSTDTSGSGQSSYSSSGQSQNTGYGTQSTPQGSGSTGGYGSSQSSQSSYGQQSSSPGYGQQPAPSSTSGSYGSSSQSSSYGQPQSGSSSQQPSYGGQQQSSGQQQSSNPPQGYGQQNQYNSSSGGGGGGGGGGNYGQDQSSMSSGGGSGGGYGNQDQSGGGGSGGYGQQASDRGGRGRGGSGGGGGGGGGGYNRSSGGYEPRGRGGGRGGRGGMGGSDRGGFNKFGGPRDQGSRHDSEQDNSDNNTIFVQGLGENVTIESVADYFKQIGIIKTNKKTGQPMINLYTDRETGKLKGEATVSFDDPPSAKAAIDWFDGKEFSGNPIKVSFATRRADFNRGGGNGRGGRGRGGPMGRGGYGGGGSGGGGRGGFPSGGGGGGGQQRAGDWKCPNPTCENMNFSWRNECNQCKAPKPDGPGGGPGGSHMGGNYGDDRRGGRGGYDRGGYRGRGGDRGGFRGGRGGGDRGGFGPGKMDSRGEHRQDRRERPY

**8. FUS(18Y-S): Full-length FUS with 18 Tyr residues substituted to Ser in the PLD**

MASNDSTQQATQSYGASPTQPGQGSSQQSSQPSGQQSYSGSSQSTDTSGSGQSSYSSSGQSQNTGSGTQSTPQGSGSTGGSGSSQSSQSSYGQQSSSPGYGQQPAPSSTSGSSGSSSQSSSSGQPQSGSSSQQPSYGGQQQSSGQQQSSNPPQGYGQQNQSNSSSGGGGGGGGGGNSGQDQSSMSSGGGSGGGYGNQDQSGGGGSGGYGQQASDRGGRGRGGSGGGGGGGGGGYNRSSGGYEPRGRGGGRGGRGGMGGSDRGGFNKFGGPRDQGSRHDSEQDNSDNNTIFVQGLGENVTIESVADYFKQIGIIKTNKKTGQPMINLYTDRETGKLKGEATVSFDDPPSAKAAIDWFDGKEFSGNPIKVSFATRRADFNRGGGNGRGGRGRGGPMGRGGYGGGGSGGGGRGGFPSGGGGGGGQQRAGDWKCPNPTCENMNFSWRNECNQCKAPKPDGPGGGPGGSHMGGNYGDDRRGGRGGYDRGGYRGRGGDRGGFRGGRGGGDRGGFGPGKMDSRGEHRQDRRERPY

**9. FUS(27Y-S): Full-length FUS with 27 Tyr residues substituted to Ser in the PLD**

MASNDSTQQATQSSGASPTQPGQGSSQQSSQPSGQQSSSGSSQSTDTSGSGQSSSSSSGQSQNTGSGTQSTPQGSGSTGGSGSSQSSQSSSGQQSSSPGSGQQPAPSSTSGSSGSSSQSSSSGQPQSGSSSQQPSSGGQQQSSGQQQSSNPPQGSGQQNQSNSSSGGGGGGGGGGNSGQDQSSMSSGGGSGGGSGNQDQSGGGGSGGSGQQASDRGGRGRGGSGGGGGGGGGGYNRSSGGYEPRGRGGGRGGRGGMGGSDRGGFNKFGGPRDQGSRHDSEQDNSDNNTIFVQGLGENVTIESVADYFKQIGIIKTNKKTGQPMINLYTDRETGKLKGEATVSFDDPPSAKAAIDWFDGKEFSGNPIKVSFATRRADFNRGGGNGRGGRGRGGPMGRGGYGGGGSGGGGRGGFPSGGGGGGGQQRAGDWKCPNPTCENMNFSWRNECNQCKAPKPDGPGGGPGGSHMGGNYGDDRRGGRGGYDRGGYRGRGGDRGGFRGGRGGGDRGGFGPGKMDSRGEHRQDRRERPY

**10. FUS(6F-G): Full-length FUS with 6 Phe residues substituted to Gly in the RBD**

AMASNDYTQQATQSYGAYPTQPGQGYSQQSSQPYGQQSYSGYSQSTDTSGYGQSSYSSYGQSQNTGYGTQSTPQGYGSTGGYGSSQSSQSSYGQQSSYPGYGQQPAPSSTSGSYGSSSQSSSYGQPQSGSYSQQPSYGGQQQSYGQQQSYNPPQGYGQQNQYNSSSGGGGGGGGGGNYGQDQSSMSSGGGSGGGYGNQDQSGGGGSGGYGQQASDRGGRGRGGSGGGGGGGGGGYNRSSGGYEPRGRGGGRGGRGGMGGSDRGGGNKGGGPRDQGSRHDSEQDNSDNNTIFVQGLGENVTIESVADYFKQIGIIKTNKKTGQPMINLYTDRETGKLKGEATVSFDDPPSAKAAIDWFDGKEFSGNPIKVSFATRRADGNRGGGNGRGGRGRGGPMGRGGYGGGGSGGGGRGGGPSGGGGGGGQQRAGDWKCPNPTCENMNFSWRNECNQCKAPKPDGPGGGPGGSHMGGNYGDDRRGGRGGYDRGGYRGRGGDRGGGRGGRGGGDRGGGGPGKMDSRGEHRQDRRERPY

**11. FUS(6Y-S): Full-length FUS with 6 Tyr residues substituted to Ser in the RBD**

AMASNDYTQQATQSYGAYPTQPGQGYSQQSSQPYGQQSYSGYSQSTDTSGYGQSSYSSYGQSQNTGYGTQSTPQGYGSTGGYGSSQSSQSSYGQQSSYPGYGQQPAPSSTSGSYGSSSQSSSYGQPQSGSYSQQPSYGGQQQSYGQQQSYNPPQGYGQQNQYNSSSGGGGGGGGGGNYGQDQSSMSSGGGSGGGYGNQDQSGGGGSGGYGQQASDRGGRGRGGSGGGGGGGGGGSNRSSGGSEPRGRGGGRGGRGGMGGSDRGGFNKFGGPRDQGSRHDSEQDNSDNNTIFVQGLGENVTIESVADYFKQIGIIKTNKKTGQPMINLYTDRETGKLKGEATVSFDDPPSAKAAIDWFDGKEFSGNPIKVSFATRRADFNRGGGNGRGGRGRGGPMGRGGSGGGGSGGGGRGGFPSGGGGGGGQQRAGDWKCPNPTCENMNFSWRNECNQCKAPKPDGPGGGPGGSHMGGNSGDDRRGGRGGSDRGGSRGRGGDRGGFRGGRGGGDRGGFGPGKMDSRGEHRQDRRERPY

**12. Taf15-SNAP: This sequence includes full-length Taf15 (unshaded), a linker that is cleavable by a TEV protease (shaded in yellow), and the SNAP tag (shaded in gray).**

MSDSGSYGQSGGEQQSYSTYGNPGSQGYGQASQSYSGYGQTTDSSYGQNYSGYSSYGQSYSQSYGGYENQKQSSYSQQPYNNQGQQQNMESSGSQGGRAPSYDQPDYGQQDSYDQQSGYDQHQGSYDEQSNYDQQHDSYSQNQQSYHSQRENYSHHTQDDRRDVSRYGEDNRGYGGSQGGGRGRGGYDKDGRGPMTGSSGGDRGGFKNFGGHRDYGPRTDADSESDNSDNNTIFVQGLGEGVSTDQVGEFFKQIGIIKTNKKTGKPMINLYTDKDTGKPKGEATVSFDDPPSAKAAIDWFDGKEFHGNIIKVSFATRRPEFMRGGGSGGGRRGRGGYRGRGGFQGRGGDPKSGDWVCPNPSCGNMNFARRNSCNQCNEPRPEDSRPSGGDFRGRGYGGERGYRGRGGRGGDRGGYGGDRSGGGYGGDRSSGGGYSGDRSGGGYGGDRSGGGYGGDRGGGYGGDRGGGYGGDRGGGYGGDRGGYGGDRGGGYGGDRGGYGGDRGGYGGDRGGYGGDRGGYGGDRSRGGYGGDRGGGSGYGGDRSGGYGGDRSGGGYGGDRGGGYGGDRGGYGGKMGGRNDYRNDQRNRPYGAPGSSSGRENLYFQGMDKDCEMKRTTLDSPLGKLELSGCEQGLHRIIFLGKGTSAADAVEVPAPAAVLGGPEPLMQATAWLNAYFHQPEAIEEFPVPALHHPVFQQESFTRQVLWKLLKVVKFGEVISYSHLAALAGNPAATAAVKTALSGNPVPILIPCHRVVQGDLDVGGYEGGLAVKEWLLAHEGHRLGKPGLG

**13. Ewsr1-SNAP: This sequence includes full-length Ewsr1 (unshaded), a linker that is cleavable by a TEV protease (shaded in yellow), and the SNAP tag (shaded in gray).**

MASTDYSTYSQAAAQQGYSAYTAQPTQGYAQTTQAYGQQSYGTYGQPTDVSYTQAQTTATYGQTAYATSYGQPPTGYTTPTAPQAYSQPVQGYGTGAYDTTTATVTTTQASYAAQSAYGTQPAYPAYGQQPAATAPTRPQDGNKPTETSQPQSSTGGYNQPSLGYGQSNYSYPQVPGSYPMQPVTAPPSYPPTSYSSTQPTSYDQSSYSQQNTYGQPSSYGQQSSYGQQSSYGQQPPTSYPPQTGSYSQAPSQYSQQSSSYGQQSSFRQDHPSSMGVYGQESGGFSGPGENRSMSGPDNRGRGRGGFDRGGMSRGGRGGGRGGMGSAGERGGFNKPGGPMDEGPDLDLGPPVDPDEDSDNSAIYVQGLNDSVTLDDLADFFKQCGVVKMNKRTGQPMIHIYLDKETGKPKGDATVSYEDPPTAKAAVEWFDGKDFQGSKLKVSLARKKPPMNSMRGGLPPREGRGMPPPLRGGPGGPGGPGGPMGRMGGRGGDRGGFPPRGPRGSRGNPSGGGNVQHRAGDWQCPNPGCGNQNFAWRTECNQCKAPKPEGFLPPPFPPPGGDRGRGGPGGMRGGRGGLMDRGGPGGMFRGGRGGDRGGFRGGRGMDRGGFGGGRRGGPGGPPGPLMEQMGGRRGGRGGPGKMDKGEHRQERRDRPYGAPGSSSGRENLYFQGMDKDCEMKRTTLDSPLGKLELSGCEQGLHRIIFLGKGTSAADAVEVPAPAAVLGGPEPLMQATAWLNAYFHQPEAIEEFPVPALHHPVFQQESFTRQVLWKLLKVVKFGEVISYSHLAALAGNPAATAAVKTALSGNPVPILIPCHRVVQGDLDVGGYEGGLAVKEWLLAHEGHRLGKPGLG
